# Shape Matters: Computational Fluid Dynamics Analysis of Epiglottis Shape Influence on Airway Collapse in Obstructive Sleep Apnea Patients

**DOI:** 10.3390/biomedicines14030553

**Published:** 2026-02-28

**Authors:** Timi Gomboc, Matjaž Hriberšek, Matej Delakorda

**Affiliations:** 1 Faculty of Mechanical Engineering, University of Maribor, Smetanova 17, 2000 Maribor, Slovenia; 2 General Hospital Celje, Oblakova ulica 5, 3000 Celje, Slovenia

**Keywords:** obstructive sleep apnea, epiglottis shape, fluid mechanics, computational fluid dynamics

## Abstract

**Background**: The study investigates the influence of epiglottis morphology on airflow dynamics and mechanical loading using computational fluid dynamics (CFD) in patients with obstructive sleep apnea (OSA), where the epiglottis may contribute to upper airway obstructions during sleep. **Methods**: A two-stage analysis was conducted: first, using a simplified airway model with two distinct epiglottis shapes (flat and curved), and second, using patient-specific 3D airway geometries derived from computed tomography (CT) scans. The simplified model enabled isolated analysis of flow-related aerodynamic forces and torques acting on the epiglottis across varying flow rates and inclination angles. **Results**: Results showed that the flat-shaped epiglottis was subjected to higher aerodynamic loads, particularly at lower flow rates, indicating increased susceptibility to collapse. These findings were corroborated by simulations on patient-specific 3D airway models. **Conclusions**: The study confirms that epiglottis morphology plays a critical role in the pathogenesis of OSA and underscores the potential of CFD for personalized assessment and treatment planning.

## 1. Introduction

Obstructive sleep apnea (OSA) is a chronic respiratory disorder caused by the repetitive obstructions of the upper airway during sleep, resulting in partial or complete airflow limitation. Consequential oxygen desaturations, hypercapnia and disrupted sleep with frequent arousals contribute to the cardiovascular, metabolic, and neurocognitive diseases [1]. Because of its high prevalence, affecting up to 27% of women and 43% of men, OSA represents a significant public health burden [2,3]. The pathophysiology of OSA is heterogenous and varies between individuals. The occurrence of obstructions is influenced not only by unfavorable anatomical conditions but also by other factors that may either stabilize or worsen the respiratory pattern, so-called OSA endotypes, including upper airway dilator muscle effectiveness, arousal threshold, and ventilatory control stability. Based on the presence and severity of each of them, along with other clinical characteristics (e.g., symptoms, comorbidities), the concept of clustering patients into more homogeneous phenotypic groups has been developed. This represents a first step toward individualized treatment approaches [4,5]. Clinical assessment of OSA patients can be very challenging, as most individuals exhibit multiple predisposing factors for upper airway obstruction, and the upper airway itself comprises several collapsible segments. Moreover, the morphology of the upper airway observed during wakefulness may differ markedly from its configuration during sleep—especially at the level of the larynx [6]. Identifying the obstruction site and collapse pattern during sleep is therefore crucial for appropriate treatment selection.

**Figure 1:**
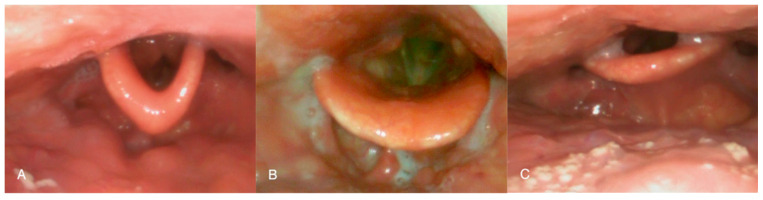
Different epiglottis shapes. Type 1—omega-shaped epiglottis (**A**); Type 2—normal concave epiglottis (**B**); and Type 3—flat epiglottis (**C**). Adapted from ref. [10].

The epiglottis is an important anatomical structure that was largely overlooked in early research on obstructive breathing disorders. However, more recent studies have demonstrated that it plays a significant role—either independently or in combination with other pharyngeal structures [7]. This has been further supported by the introduction of routine pre-operative assessments, particularly drug-induced sleep endoscopy (DISE), which has revealed that partial or complete obstruction at the level of the epiglottis occurs in approximately 20% to 40% of OSA patients [8,9]. Although it was long believed that such obstructions could not be suspected based solely on awake examination, careful observation may provide clues regarding epiglottic collapse. In a previous study, obstructions at this level were shown to occur more frequently in patients with a flat-shaped epiglottis—Figure 1C [10].

The influence of airflow on the collapse of soft tissues in the upper respiratory tract is a complex process that classical clinical methods, such as visual inspection, can only partially elucidate. Our understanding has been enhanced by dynamic radiological techniques—namely, dynamic magnetic resonance imaging (MRI), computed tomography (CT), and DISE—which provide real-time insights into changes in the shape, position, and spatial relationships of various anatomical structures. A significant advancement in this field has been the application of computational fluid dynamics (CFD). By numerically solving the Navier–Stokes equations, CFD enables detailed analysis of airflow patterns and pressure distributions within anatomically complex airway geometries, accounting for both laminar and turbulent flow conditions. Over the past few decades, CFD has been widely employed to investigate various aspects of pulmonary airflow, including ventilation efficiency, airway obstructions, and aerosol drug deposition [11].

The importance of CFD in respiratory research was further underscored during the COVID-19 pandemic, when understanding aerosol transport became a critical concern. Researchers employed CFD models to analyze the movement of virus-laden droplets through the respiratory tract—from oral or nasal inhalation to deep lung deposition [12]. These studies provided valuable insights into the risks of airborne transmission and the effectiveness of protective measures such as face masks and ventilation systems. In parallel, CFD simulations played a key role in optimizing aerosol-based drug delivery, enhancing targeted deposition within the lungs for the treatment of respiratory infections [13]. CFD has also enabled detailed visualization of airflow fields within lumens of varying shapes, providing a foundation for analyzing the mechanical impact of flow on anatomical structures of the upper airway [14,15,16]. This modeling approach has proven useful in predicting the severity of sleep-disordered breathing and assessing the potential success of surgical interventions [17].

A key shortcoming of the given approach is its technical complexity, primarily due to the requirement for precise input data, including detailed characteristics of the tissues under study. By integrating high-resolution imaging techniques such as CT and MRI, modern CFD models have achieved unprecedented accuracy in replicating patient-specific airway geometries. This advancement has enabled more precise assessments of airflow dynamics, particularly in conditions like asthma, chronic obstructive pulmonary disease (COPD), and acute respiratory infections. Moreover, the growing application of CFD in personalized medicine underscores its potential for tailoring treatments based on individual anatomical and physiological characteristics [12].

In the presented research, we investigated how the shape of the epiglottis influences the pressure field within the airway and, consequently, the aerodynamic forces acting upon it that may lead to collapse. To explore this, numerical simulations were conducted for two distinct epiglottis shapes—flat and curved. The simulations were first performed using a simplified airway lumen geometry to allow a direct comparison of shape effects under identical flow conditions. Subsequently, simulations were carried out using patient-specific upper airway geometries derived from CT scans, providing deeper insight into the interaction between individual airflow dynamics and epiglottis morphology.

## 2. Materials and Methods

Because real human airways are unique, it is difficult to perform parametric studies under unifying flow conditions to identify the critical parameters and compare different patient-related geometrical cases. For this reason, the research was conducted in two steps: The first was based on designing a simplified numerical experiment, where two different epiglottis shapes were positioned into a pipe, representing the airway, with matching diameter and length. This decision provides the same upstream flow conditions with only epiglottis shape affecting the local flow conditions, enabling a focused study of the effect of epiglottis shape on OSA conditions. In the second step of the research, two real human airways with different epiglottis shapes were analyzed. In the following, basic steps in constructing the 3D models based on patient-specific CT scans are described first, followed by a description of the two cases with simplified airway models.

### 2.1. Geometrical Models

#### 2.1.1. CT Scan-Based Patient-Specific Geometrical Models

**Figure 2:**
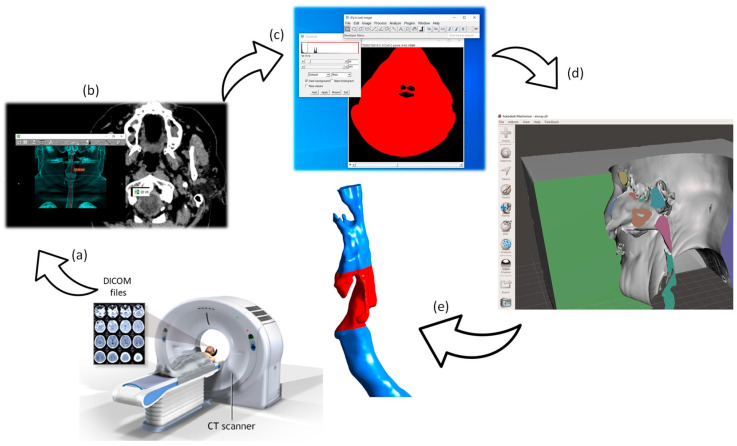
Workflow from CT imaging to the creation of a 3D model of the upper airway: CT scan (**a**,**b**); image review using RadiAnt DICOM Viewer (**c**); airway morphology extraction from the image set using Fiji software (**d**); 3D model processing in Meshmixer (Autodesk, version 3.5) (**e**).

For physiologically accurate CFD analysis, obtaining realistic anatomical geometries of the respiratory tract and the epiglottis is essential. High-resolution CT scans provide detailed 3D reconstructions, enabling precise simulations that reflect real physiological conditions. The workflow from CT scan to the 3D reconstruction is presented in Figure 2.

**Figure 3:**
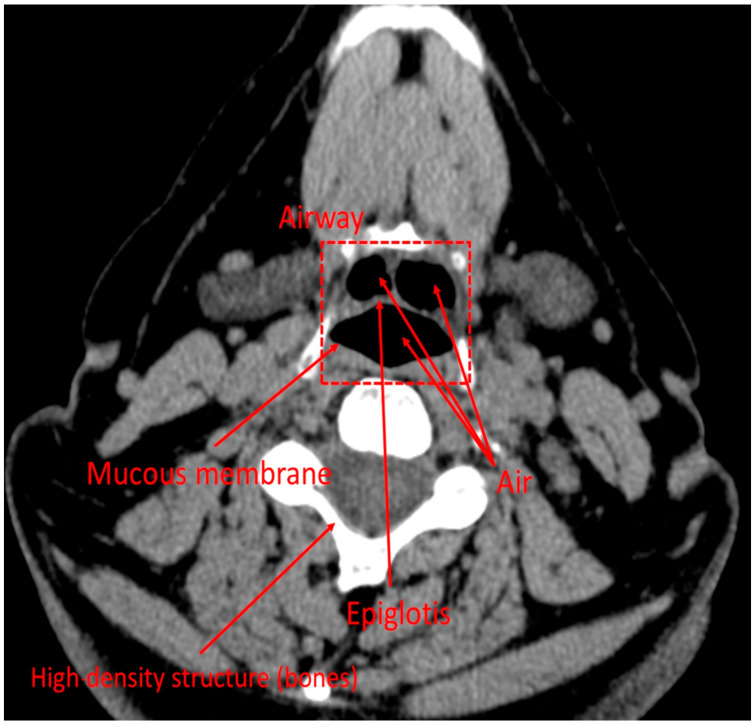
CT image of the supraglottic region in the axial plane.

First, an anonymized CT scan captures cross-sectional images, stored in DICOM format, containing both imaging data and patient metadata. RadiAnt DICOM viewer was used to analyze image properties, including pixel dimensions. Airways appear dark on CT images due to the low X-ray attenuation of air, whereas soft tissues appear in shades of gray and bone appears white (Figure 3). To isolate airways, Fiji software (version 2.16.0) was employed. The air–mucous membrane boundary, seen as a transition between dark air-filled spaces and grayish soft tissue, was identified using the threshold function. Unwanted gaps were filled using the Flood fill function, ensuring that only airway structures remained present.

**Figure 4:**
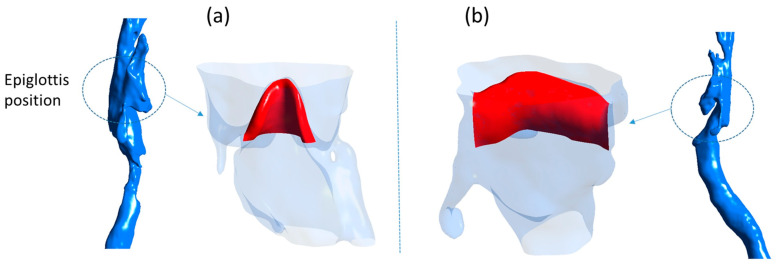
Real human upper airway with curved epiglottis shape (**a**) and flat epiglottis shape (**b**).

Next, the invert function was applied to define the airways for 3D modeling, preventing the surroundings from being modeled instead. The 3D Viewer function then converted the image set into an STL file. Finally, Meshmixer software was used for adjustments, resulting in two 3D airway models, as shown in Figure 4.

The two 3D models reconstructed from CT images differ in the shape of the epiglottis. Figure 4a shows a curved epiglottis, whereas Figure 4b depicts a flat epiglottis. The cross-sectional airflow areas between the two shapes are very similar, with a difference of less than 1%.

Once the 3D model was generated, a polyhedral mesh with five boundary layers was created to optimize the computational efficiency and accuracy of the resolution of the near-wall regions, a key element when applying the k-ω SST turbulence model in CFD simulations). Ansys Fluent [6] with Fluent meshing was used for mesh generation and all further numerical simulations. The computational mesh of the curved epiglottis case consisted of 9,767,310 cells, and the mesh of the flat epiglottis case consisted of 9,866,854 cells.

#### 2.1.2. Simplified Geometrical Model

**Figure 5:**
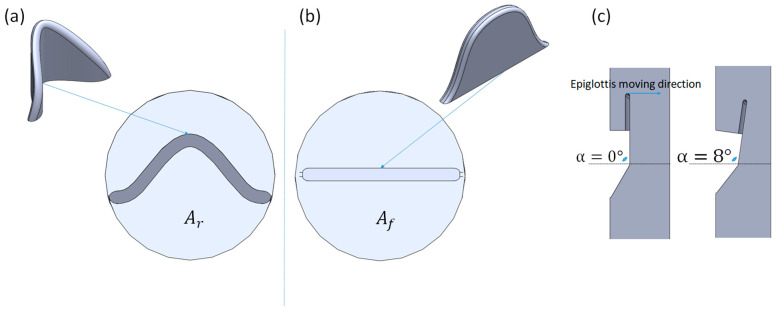
Geometrical model of curved epiglottis shape (**a**), and flat epiglottis shape (**b**). Epiglottis rotation (inclination) angle and cross-section areas (**c**). The arrows indicate the possible direction of change in the angle determining the position of the epiglottis.

In the case of the simplified airways, a straight pipe with the epiglottis forming the flow obstruction was created. The total length of the pipe was 150 mm, with a diameter of 40 mm, and the epiglottis positioned in the middle of the pipe. Both selected typical epiglottis geometries are presented in Figure 5.

For each epiglottis shape, eleven numerical simulations were performed. The inclination angle of the epiglottis relative to the incoming airflow was varied between 0° and 15°, as shown in Figure 5. The channel cross-sectional area at the base of the epiglottis was kept the same for both epiglottis shapes ($A_{r}=A_{f}$) for initial epiglottis inclination angle 0°. With the position of the inclination angle of the epiglottis changing for each computed case, the cross-section areas ($A_{r}\;and\;A_{f}$) also changed, which consequently means that the local duct geometry was different for both epiglottis shapes. Given that the airflow through the tube is kept constant, the change in the cross-sectional area affects the pressure and velocity distributions and consequently the aerodynamic force acting on the epiglottis. In this way the influence of the epiglottis shape can be evaluated under unified test conditions, making the comparison more decisive.

### 2.2. Numerical Model and Boundary Conditions

The CFD model for simulating respiratory airflow and particle transport is based on solving the Reynolds-averaged Navier–Stokes equations, governing the flow of incompressible air. In the current case, the Ansys Fluent CFD software (version 25.2) was used. Because the airflow in the airways is turbulent, a suitable turbulence model was required. We selected the k–ω Shear Stress Transport (SST) model, as it provides a good balance between computational cost and accuracy. As in our previous detailed CFD study of particle tracking under turbulent respiratory flow conditions, the numerical model used in this work was validated against experimental data [12].

With respect to the boundary conditions at the inlet, a pressure boundary condition was applied, specifying a static pressure of 0 Pa. Since the study simulates human breathing, the inspiratory and expiratory volume flow rates can be described using a sinusoidal function. However, as obstructive events occur during inspiration, when the aerodynamic force acts in the direction of the main airflow, only the inspiratory phase was considered in this analysis. The maximum inspiratory volumetric flow rate was 25 L/min, which corresponded to the highest magnitude of the resulting aerodynamic force. The selected inspiratory flow rates (5–25 L/min) were chosen to reflect physiologically relevant peak inspiratory conditions during sleep in adults, encompassing both flow-limited breathing and near-normal inspiratory airflow observed in patients with OSA. Therefore, a constant mass flow outlet boundary condition was applied during the CFD simulation. For a flow rate of 25 L/min, the corresponding mass flow rate was 4.85 × 10^−4^ kg/s, which was prescribed as the outlet boundary condition. The air inlet temperature was set to 30 °C and held constant across all simulated test cases.

In order to analyze the results and compare the effect of different epiglottis shapes, several parameters were evaluated. First, the magnitude and direction of the aerodynamic force $F_{aero}$, exerted by the flow on the epiglottis, was calculated from obtained flow results as follows:

$$F_{aero}=\sum \left(-p_{i}\cdot n_{i}+\tau_{i}\right)\cdot S_{i}$$

$$M_{aero}=\sum r_{i}\times \left(-p_{i}\cdot n_{i}+\tau_{i}\right)\cdot S_{i}$$

with the summation of the pressure (pi) and shear stress ($\tau_{i}$) contributions over the surface element sizes Si, comprising the epiglottis shape. The resulting aerodynamic torque $M_{aero}$, acting on the epiglottis, was defined with respect to the base point of the epiglottis.

The influence of the pressure field was also evaluated by means of the epiglottis flow resistance (Rp), defined as follows:

$$R_{p}=\frac{\Delta p}{\dot{V}}$$

where $\dot{V}$ is the volumetric low rate and Δp is the pressure drop along the constriction area, defined as the difference between the average pressure values at the inlet and outlet of the constriction area.

## 3. Results

### 3.1. Simplified Geometrical Model

The simplified geometrical model was used for a computational study of the influence of the epiglottis shape on the flow conditions and resulting aerodynamic force and torque, acting on the epiglottis. By making a geometrically simple model, the development of several airway geometries was possible, resembling a quasi-static epiglottis condition, that forms during the epiglottis movement and finally collapses into position, effectively blocking the airflow in the upper airways. Different angles of the epiglottis inclination also have an effect on the magnitude of the flow cross-sectional area, with the latter decreasing with increasing rotation (i.e., inclination angle) of the epiglottis. In the case of the flat epiglottis, the decrease in cross-sectional area is more pronounced than in the curved shape. The selected geometrical configuration and the performed computational parametric study therefore enable a clear evaluation of the influence that both the geometry and position of the epiglottis have on the occurrence of obstructive events.

**Figure 6:**
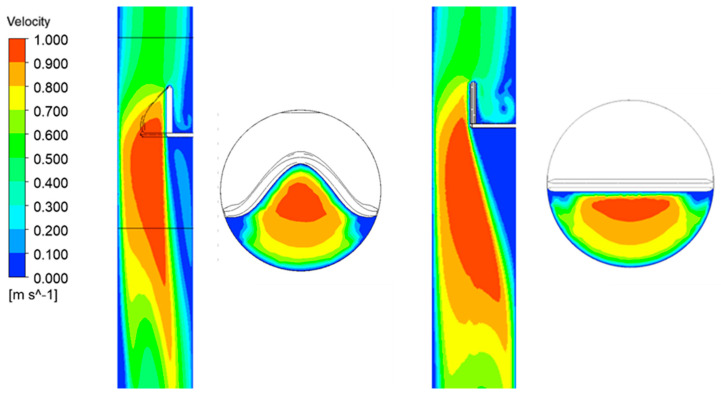
Velocity fields in the cross-section along the airway for curved (**left**) and flat epiglottis (**right**).

Figure 6 presents the velocity fields in cross-sections along the airway for both geometrical cases. Because the initial cross-sectional area behind the epiglottis is identical (Ar = Af), the flow conditions are quite similar in both shapes. When the epiglottis is rotated to a new position (as shown in Figure 5 for α = 8°), the cross-sectional area behind the epiglottis decreases, resulting in changes in both the pressure and velocity fields.

**Figure 7:**
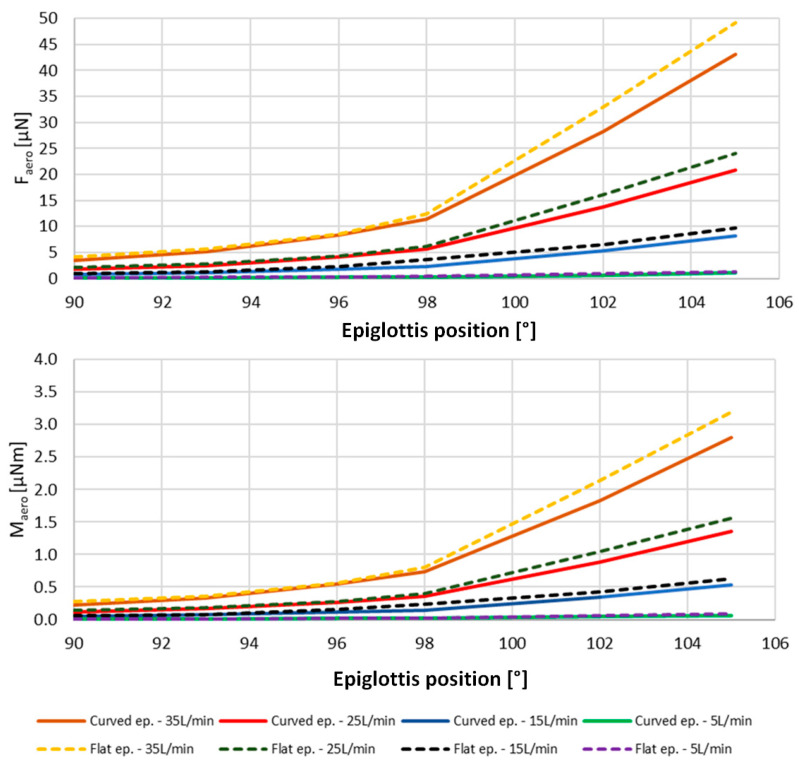
Aerodynamic force and resulting torque exerted on the epiglottis surface at different epiglottis positions and different flow rates—the simplified geometry case.

The cross-sectional area behind the epiglottis decreases more rapidly in the case of the flat epiglottis, leading to significant changes in aerodynamic force and torque values, as shown in Figure 7, as well as alterations in flow resistance. Increasing the position angle—that is, moving the epiglottis closer to the adjacent posterior pharyngeal wall—results in a substantial increase in airflow velocity and a corresponding pressure drop. This, in turn, produces a considerable pressure difference across the epiglottis surface and along the constriction region.

As the rotation angle approaches α = 15°, the difference in flow resistance between the flat and curved epiglottis becomes more pronounced. At the highest rotation angle, the flow resistance of the flat epiglottis is nearly three times greater than that of the curved shape. Regarding mechanical loads on the epiglottis, the resulting aerodynamic force—calculated by integrating the pressure and shear stress distributions along the epiglottis surface—follows the same trend as flow resistance, with force values increasing as the rotation angle increases. Given that determining the exact flow rate in a realistic patient case is challenging, the influence of varying flow rates on force and torque loading was analyzed in a simplified geometric model, as presented in Figure 7. At the lowest flow rate of 5 L/min, both force and torque loadings are approximately 25% higher for the flat epiglottis compared to the curved one. At higher flow rates, this difference slightly decreases to about 18% for the maximum computed flow rate, although the absolute values of force and torque increase quadratically with flow rate. Therefore, in sleep-related scenarios involving lower airflow rates, the flat epiglottis is more susceptible to flow-induced mechanical loading, making airway obstruction more likely. Overall, the flat epiglottis consistently represents the less favorable shape across all tested conditions, as further confirmed by the analysis of realistic 3D geometries.

### 3.2. The Realistic 3D Models

Since simulations with realistic airway geometries required significantly longer computational times, the key findings from the simplified geometry were validated by performing additional simulations using two realistic airway models. These models incorporated anatomically derived epiglottis shapes and positions, with varying airflow rates to mimic different breathing scenarios.

**Figure 8:**
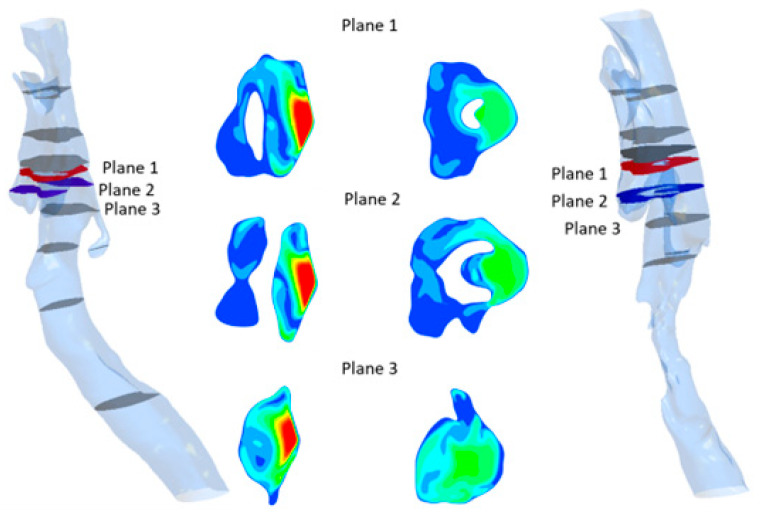
The absolute velocity field for the case of 25 L/min, realistic geometry with flat epiglottis (**left**) and curved epiglottis (**right**).

**Figure 9:**
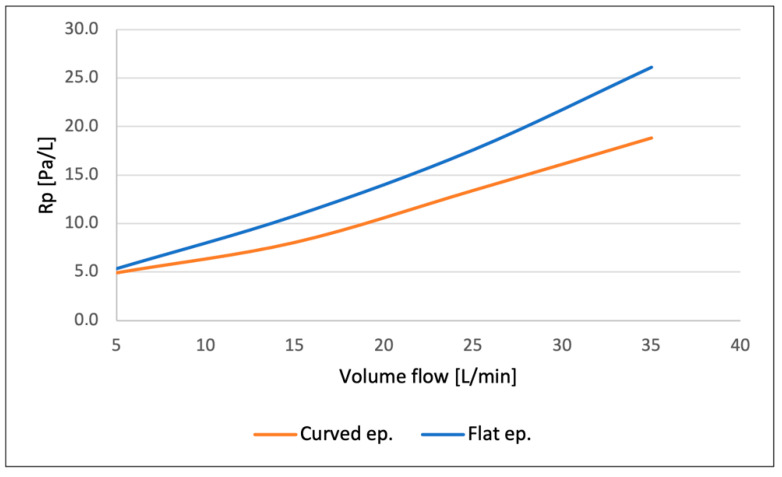
The flow resistance values for the case of the real geometry and different flow rates.

The flow characteristics at a breathing rate of 25 L/min are illustrated in Figure 8, showing absolute velocity values at selected cross-sections near the epiglottis. At the narrowest cross-section—formed between the epiglottis and the airway walls—the velocity magnitude is higher for the flat epiglottis than for the curved one. Figure 9 presents the flow resistance values as a function of volumetric flow rate, clearly indicating that the flat epiglottis produces substantially higher flow resistance compared to the curved shape, as a consequence of larger local pressure differences.

**Figure 10:**
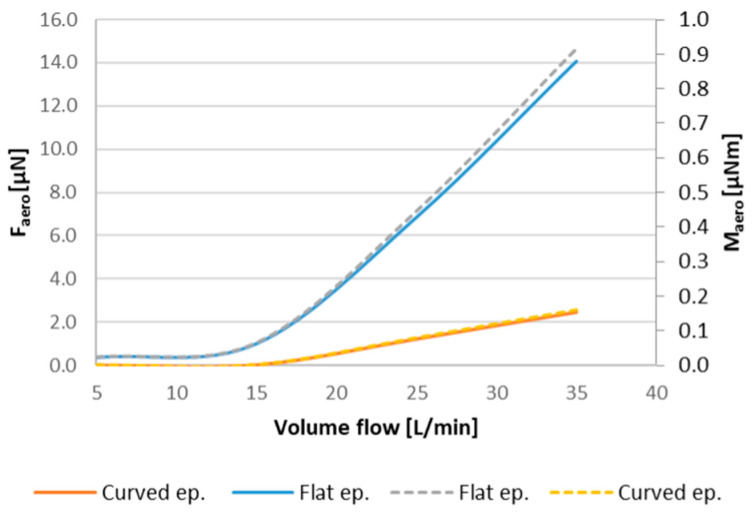
The resulting force (solid lines) and torque (dashed lines) exerted on the epiglottis surface at different flow rates for the real geometry case.

From the perspective of mechanical energy conservation in the airflow, this implies a more pronounced local pressure drop downstream of the epiglottis for the flat shape, resulting in a higher aerodynamic force acting on the epiglottis (Figure 10). This increased aerodynamic force, in turn, generates a greater torque on the epiglottis, promoting its deformation and, in severe cases, potentially leading to complete airway obstruction.

## 4. Discussion

Given the extensive use of CFD in the modeling of pulmonary airflow, its application extends naturally to understanding the role of the larynx in respiratory mechanics, where the epiglottis plays a key role. Using patient-specific 3D modeling, the CFD has provided an additional understanding of airway collapse mechanisms and of quantifying the severity of anatomical airway restrictions in OSA patients [18,19,20]. A statistically significant correlation was found between CFD-derived airway resistance and OSA severity measured by the apnea–hypopnea index. Importantly, according to previous findings, the location of flow limitation and airway collapse are not always identical [21]. Therefore, CFD analysis may serve as a valuable tool for guiding the selection of targeted therapeutic interventions in OSA management.

The relations between structures in the pharynx and larynx may be considerably different in the awake state and during sleep. To date, several morphological characteristics have been described in the population of patients with sleep breathing disorders, by which such patients differ from healthy individuals; however, the authors mostly focused on the shape of soft tissue in the oropharynx and relations between them. Some other studies have shown that the frequency of epiglottis obstructions depends on the relationship between the base of the tongue and the lingual side of the epiglottis, or its displacement towards the back wall of the pharynx [22]. Similar conclusions were reached in a study in which they analyzed the influence of vocal cord visualization—the Cormack–Lehane score on the severity of OSA [23]. According to the aforementioned research, the obstructions at the level of the epiglottis are probably influenced by both the epiglottis position that determines the cross-sectional area of the lumen behind it and the shape of the epiglottis itself.

Within the current endotypic model of OSA, epiglottic shape may be considered in relation to both anatomical and neuromuscular factors. From a clinical perspective, epiglottic shape can be readily assessed during awake examination, making it a practical anatomical marker. However, it should not be interpreted as a purely rigid or static feature. The epiglottis is composed of elastic cartilage and is mechanically coupled to surrounding structures through ligamentous and soft tissue attachments, most notably the hyoepiglottic ligament, which links it to the hyoid bone and the tongue base. Consequently, its observed shape and position may reflect not only intrinsic structural characteristics but also the biomechanical influence of adjacent structures and their neuromuscular control. Within this framework, epiglottic morphology may therefore primarily represent an anatomical determinant of airway collapsibility, while also indirectly reflecting aspects of upper airway dilator muscle responsiveness. This structural–neuromuscular interaction may be reflected in the dynamic collapse patterns observed during drug-induced sleep endoscopy (DISE), where epiglottic behavior often changes in response to tongue base position and airway configuration.

Obstructions at the pharyngeal level typically progress gradually, with the cross-sectional area decreasing as respiratory effort (i.e., downstream pressure) increases, until the point of critical closing pressure is reached and the airway lumen collapses, resulting in complete cessation of airflow. In contrast, obstructions at the level of the epiglottis in the anteroposterior direction occur abruptly [24]. When the negative pressure difference increases to a critical value in the lumen behind the laryngeal surface of the epiglottis, it acts as a one-way valve, and sudden closure occurs with the airflow being completely interrupted—a mechanism known as the “trap-door phenomenon”.

From an engineering perspective, the human upper airway can be likened to a collapsible tube, where reductions in cross-sectional area—particularly in the region of the epiglottis—significantly affect airflow behavior. Inspiratory airflow patterns vary among individuals due to differences in age, physical condition, and the presence of respiratory diseases. Among these factors, varying inspiratory flow rates in combination with different epiglottic morphologies can create conditions that favor the occurrence of OSA, as demonstrated in this study. Specifically, the resulting torque on the flat epiglottis can be up to 25% higher than that observed for the curved epiglottis under the same flow rate and epiglottis position. Although only a limited range of epiglottis positions was numerically analyzed for the simplified geometry, the obtained results clearly show a trend of increasing mechanical loads on the flat configuration compared with the curved one. The resulting torques on the epiglottis can contribute to the onset of OSA, particularly because, with increasing epiglottis rotation (i.e., inclination angle), the magnitude of the resulting torque rises markedly, creating conditions that accelerate epiglottic collapse.

Since the epiglottis was modeled as rigid in the present study, it is important to assess the implications of this assumption for a physiologically more realistic flexible configuration. An increase in epiglottic tissue flexibility is expected to lower the critical flow rate associated with the onset of obstruction compared with a more rigid epiglottis. Consequently, the rigid-body approximation adopted in the present computational study may be interpreted as representing an upper-bound scenario in terms of resistance to airway collapse and, thus, to the onset of OSA.

The results of the current analysis indicate that, within the rigid epiglottis framework, a flat epiglottic geometry is more susceptible to collapse than a curved configuration. It is therefore reasonable to anticipate that this relative susceptibility would persist in a fluid–structure interaction (FSI) analysis incorporating tissue flexibility. While the absolute rotational displacements of the epiglottis would differ between rigid and deformable models, the initial phase of motion—dominated primarily by global displacement—would be expected to remain comparable. As deformation progresses and bending effects become significant, structural compliance would increasingly influence the fluid–structure interaction, indicating that the system is approaching the collapse regime.

Accordingly, although the rigid-body assumption neglects deformation-induced effects, the present results provide valuable insight into the relative stability of different epiglottic shapes and may serve as a meaningful indicator of their susceptibility to the onset of OSA.

The findings of the present study underscore CFD as a valuable tool for biomechanical investigation of airway collapse in OSA. The results are consistent with previous clinical observations demonstrating that epiglottic obstruction occurs more frequently in patients with a flat epiglottis configuration [10], and provide a biomechanical explanation for this association through increased aerodynamic force, torque, and flow resistance. Rather than suggesting routine CT-based assessment in OSA patients, the findings support careful evaluation of epiglottic shape during standard clinical examination and DISE. In this context, biomechanical modeling may enhance interpretation of collapse patterns, improve risk stratification, and support more targeted therapeutic and surgical decision-making.

However, the patient-specific validation was based on only two airway geometries and should therefore be interpreted as proof-of-concept evidence rather than statistical confirmation. In addition, the epiglottis was modeled as a rigid structure and neuromuscular influences were not explicitly incorporated, which limits direct extrapolation to in vivo dynamic collapse behavior. Despite these limitations, the developed CFD models enable detailed quantification of mechanical loading and may, in the future, contribute to more individualized assessment and treatment planning. Larger cohort studies and fluid–structure interaction analyses will be required to further establish the clinical relevance of epiglottic morphology as an anatomical determinant of airway vulnerability in OSA.

## 5. Conclusions

This study demonstrates that epiglottis morphology significantly influences airflow characteristics and the associated mechanical loading in computational models of OSA. In both simplified and patient-specific airway geometries, the flat epiglottis was associated with higher aerodynamic forces, torque, and flow resistance compared with the curved configuration, particularly under inspiratory flow conditions typical of sleep.

Although the rigid-body assumption limits direct extrapolation to in vivo collapse behavior, the findings suggest that epiglottic shape may contribute to differences in biomechanical stability under airflow. These computational findings are consistent with previously reported clinical observations. Overall, the results support consideration of epiglottis morphology as a potentially relevant anatomical factor in airway vulnerability and highlight the value of CFD modeling for mechanistic investigation of upper airway dynamics.

## Data Availability

Dataset available on request from the authors.

## References

[1] Gleeson M., McNicholas W.T. (2022). Bidirectional relationships of comorbidity with obstructive sleep apnoea. Eur. Respir. Rev..

[2] Peppard P.E., Young T., Barnet J.H., Palta M., Hagen E.W., Hla K.M. (2013). Increased Prevalence of Sleep-Disordered Breathing in Adults. Am. J. Epidemiol..

[3] Heinzer R., Vat S., Marques-Vidal P., Marti-Soler H., Andries D., Tobback N., Mooser V., Preisig M., Malhotra A., Waeber G. (2015). Prevalence of sleep-disordered breathing in the general population: The HypnoLaus study. Lancet Respir. Med..

[4] Malhotra A., Mesarwi O., Pepin J.-L., Owens R.L. (2020). Endotypes and phenotypes in obstructive sleep apnea. Curr. Opin. Pulm. Med..

[5] Eckert D.J. (2018). Phenotypic approaches to positional therapy for obstructive sleep apnoea. Sleep Med. Rev..

[6] Campanini A., Canzi P., De Vito A., Dallan I., Montevecchi F., Vicini C. (2010). Awake versus sleep endoscopy: Personal experience in 250 OSAHS patients. Acta Otorhinolaryngol. Ital..

[7] Torre C., Camacho M., Liu S.Y., Huon L., Capasso R. (2015). Epiglottis collapse in adult obstructive sleep apnea: A systematic review. Laryngoscope.

[8] Fernández-Julián E., Pérez G., Ángel M., García-Callejo J., Ferrer F., Martí F., Marco J. (2014). Surgical planning after sleep versus awake techniques in patients with obstructive sleep apnea. Laryngoscope.

[9] Ravesloot M.J.L., de Vries N. (2011). One hundred consecutive patients undergoing drug-induced sleep endoscopy: Results and evaluation. Laryngoscope.

[10] Delakorda M., Ovsenik N. (2018). Epiglottis shape as a predictor of obstruction level in patients with sleep apnea. Sleep Breath..

[11] Zhao J., Feng Y., Fromen C.A. (2020). Glottis motion effects on the particle transport and deposition in a subject-specific mouth-to-trachea model: A CFPD study. Comput. Biol. Med..

[12] Wedel J., Steinmann P., Štrakl M., Hriberšek M., Cui Y., Ravnik J. (2022). Anatomy matters: The role of the subject-specific respiratory tract on aerosol deposition—A CFD study. Comput. Methods Appl. Mech. Eng..

[13] Wang Y., Jin Z., Cui Y., Dong R., Li L., Lizal F., Hriberšek M., Ravnik J., Yang M., Liu Y. (2024). An individualised 3D computational flow and particle model to predict the deposition of inhaled medicines — A case study using a nebuliser. Comput. Methods Programs Biomed..

[14] Jeong S.-J., Kim W.-S., Sung S.-J. (2006). Numerical investigation on the flow characteristics and aerodynamic force of the upper airway of patient with obstructive sleep apnea using computational fluid dynamics. Med. Eng. Phys..

[15] Luo H., Sin S., McDonough J.M., Isasi C.R., Arens R., Wootton D.M. (2014). Computational fluid dynamics endpoints for assessment of adenotonsillectomy outcome in obese children with obstructive sleep apnea syndrome. J. Biomech..

[16] Zarandi M.A.F., Garman K., Rhee J.S., Woodson B.T., Garcia G.J. (2021). Effect of tube length on the buckling pressure of collapsible tubes. Comput. Biol. Med..

[17] Hsieh S.-W., Lai C.-L., Liu C.-K., Hsieh C.-F., Hsu C.-Y. (2012). Obstructive sleep apnea linked to wake-up strokes. J. Neurol..

[18] Liu Y., Mitchell J., Chen Y., Yim W., Chu W., Wang R.C. (2018). Study of the upper airway of obstructive sleep apnea patient using fluid structure interaction. Respir. Physiol. Neurobiol..

[19] Pirnar J., Dolenc-Grošelj L., Fajdiga I., Žun I. (2015). Computational fluid-structure interaction simulation of airflow in the human upper airway. J. Biomech..

[20] Mihaescu M., Murugappan S., Gutmark E., Donnelly L.F., Kalra M. (2008). Computational Modeling of Upper Airway Before and After Adenotonsillectomy for Obstructive Sleep Apnea. Laryngoscope.

[21] Yanagisawa-Minami A., Sugiyama T., Iwasaki T., Yamasaki Y. (2020). Primary site identification in children with obstructive sleep apnea by computational fluid dynamics analysis of the upper airway. J. Clin. Sleep Med..

[22] Marques M., Genta P.R., Sands S.A., Azarbazin A., de Melo C., Taranto-Montemurro L., White D.P., Wellman A. (2017). Effect of Sleeping Position on Upper Airway Patency in Obstructive Sleep Apnea Is Determined by the Pharyngeal Structure Causing Collapse. Sleep.

[23] Bolzer A., Toussaint B., Rumeau C., Gallet P., Jankowski R., Nguyen D.T. (2019). Can anatomical assessment of hypopharyngolarynx in awake patients predict obstructive sleep apnea?. Laryngoscope.

[24] Azarbarzin A., Marques M., Sands S.A., de Beeck S.O., Genta P.R., Taranto-Montemurro L., de Melo C.M., Messineo L., Vanderveken O.M., White D.P. (2017). Predicting epiglottic collapse in patients with obstructive sleep apnoea. Eur. Respir. J..

